# Individual differences in bodily freezing predict emotional biases in decision making

**DOI:** 10.3389/fnbeh.2014.00237

**Published:** 2014-07-03

**Authors:** Verena Ly, Quentin J. M. Huys, John F. Stins, Karin Roelofs, Roshan Cools

**Affiliations:** ^1^Behavioural Science Institute, Radboud University NijmegenNijmegen, Netherlands; ^2^Donders Institute for Brain, Cognition, and Behaviour, Centre for Cognitive Neuroimaging, Radboud University NijmegenNijmegen, Netherlands; ^3^Translational Neuromodeling Unit, Institute for Biomedical Engineering, University of Zurich and ETH ZurichZurich, Switzerland; ^4^Department of Psychiatry, Psychotherapy and Psychosomatics, Hospital of Psychiatry, University of ZurichZurich, Switzerland; ^5^Faculty of Human Movement Sciences, MOVE Research Institute Amsterdam, VU University AmsterdamAmsterdam, Netherlands; ^6^Department of Psychiatry, Radboud University Nijmegen Medical CentreNijmegen, Netherlands

**Keywords:** emotion, freezing, decision making, learning, approach-avoidance

## Abstract

Instrumental decision making has long been argued to be vulnerable to emotional responses. Literature on multiple decision making systems suggests that this emotional biasing might reflect effects of a system that regulates innately specified, evolutionarily preprogrammed responses. To test this hypothesis directly, we investigated whether effects of emotional faces on instrumental action can be predicted by effects of emotional faces on bodily freezing, an innately specified response to aversive relative to appetitive cues. We tested 43 women using a novel emotional decision making task combined with posturography, which involves a force platform to detect small oscillations of the body to accurately quantify postural control in upright stance. On the platform, participants learned whole body approach-avoidance actions based on monetary feedback, while being primed by emotional faces (angry/happy). Our data evidence an emotional biasing of instrumental action. Thus, angry relative to happy faces slowed instrumental approach relative to avoidance responses. Critically, individual differences in this emotional biasing effect were predicted by individual differences in bodily freezing. This result suggests that emotional biasing of instrumental action involves interaction with a system that controls innately specified responses. Furthermore, our findings help bridge (animal and human) decision making and emotion research to advance our mechanistic understanding of decision making anomalies in daily encounters as well as in a wide range of psychopathology.

Emotions are well-known to influence decision making (Damasio et al., 1996; Damasio, 1997; Roelofs et al., 2009a,b, 2010b; Von Borries et al., 2012). Such emotional biases may reflect interactions between distinct (e.g., emotional vs. deliberative) systems for behavioral control (Kahneman and Frederick, 2002; Camerer et al., 2005). However, the precise nature of these interactions remains unclear. One possibility is that emotional biasing of action selection reflects an interaction between a Pavlovian and an instrumental control system (Dayan et al., 2006; Seymour and Dolan, 2008). According to this view, emotional biasing of action selection might reflect effects of a Pavlovian system that regulates innately specified, evolutionarily preprogrammed responses to reward- or punishment-predictive stimuli. The power of this Pavlovian system is illustrated, for example, by the inability of chicks to learn to run away from a food cup in order to obtain food (Hershberger, 1986). Thus, the innately specified tendency to approach the food prevented acquisition of the instrumental run-away response. In humans, Pavlovian influences are evidenced by a series of experiments (Crockett et al., 2009; Guitart-Masip et al., 2011, 2012, 2014; Cavanagh et al., 2013) crossing motivational valence (appetitive vs. aversive) and action (behavioral activation vs. behavioral inhibition) showing that reward potentiates behavioral activation, while punishment potentiates behavioral inhibition (see also, Carver and White, 1994; Gray and McNaughton, 2000, where close reward-activation and punishment-inhibition couplings have been suggested). Similar Pavlovian response tendencies have been studied extensively using Pavlovian-to-instrumental transfer paradigms in animals, as well as humans, where reward- or punishment-predictive stimuli modulate instrumental responses (Estes and Skinner, 1941; Estes, 1948; Di Giusto et al., 1974; Lovibond, 1983; Dickinson and Balleine, 1994; Bray et al., 2008; Talmi et al., 2008; Declercq and De Houwer, 2009; Trick et al., 2011; Prévost et al., 2012; Geurts et al., 2013; Lewis et al., 2013; Lovibond et al., 2013). Although it has been shown before that basic learning mechanisms may be relevant to understanding human social interactions (Olsson et al., 2005), the hypothesis that Pavlovian-like response tendencies account for emotional biasing of action selection by emotional faces has never been tested. Elucidating such relationship is important to advance our mechanistic understanding of decision making anomalies that play a critical role in daily encounters as well as in (social) psychopathologies.

The main aim of this study was to test directly the hypothesis that effects of emotional faces on action selection reflect effects of a system that regulates innately specified responses. To this end, we investigated whether effects of emotional faces on approach and avoidance responses can be predicted by effects of emotional faces on bodily freezing, an innately specified response to aversive vs. appetitive cues. Specifically, we predicted that effects of angry vs. happy faces on instrumental avoidance vs. approach responses would be predicted by effects of angry vs. happy faces on bodily freezing.

Bodily freezing, one of the most widely recognized defensive reactions to threat (Blanchard et al., 2001), can be reliably measured in humans using posturography (Carpenter et al., 1999; Azevedo et al., 2005; Stins and Beek, 2007; Roelofs et al., 2010a). Using a force-platform, small bodily oscillations can be accurately detected to quantify postural control in upright stance when watching emotional pictures. Using this method, we have previously shown that bodily freezing to aversive pictures is exacerbated in anxious (Roelofs et al., 2010a) and traumatized (Hagenaars et al., 2014) individuals. Moreover, bodily freezing has been shown to be associated with bradycardia, i.e., reductions in heartrate, which is a well accepted indicator of human freezing (Lang et al., 2000; Marx et al., 2008; Roelofs et al., 2010a; Hermans et al., 2013). Combining posturography with a novel emotional decision making task allowed us to quantify the innately specified response to the emotional face, i.e., bodily freezing, as well as its predictive value for the subsequent emotional biasing effect on the instrumental response.

Our second aim was to test the hypothesis that the interaction between emotion and action selection occurs at the level of motivational valence rather than at the level of motor output. This question parallels a debate in the Pavlovian-to-instrumental transfer literature (Lovibond, 1983), where recent work in humans (Huys et al., 2011) has suggested that such Pavlovian-to-instrumental transfer occurs at the level of motivational valence rather than motor output. Huys et al. (2011) have shown that appetitive Pavlovian cues do not potentiate all “go”-responses, but only those “go”-responses that are labeled as “approach”. In fact, “go”-responses labeled as avoidance were suppressed by appetitive cues. Conversely, aversive Pavlovian cues potentiated “avoid-go”-responses, while suppressing “approach-go”-responses. This action-specificity indicates that Pavlovian-to-instrumental transfer is unlikely to reflect interaction at the level of motor output. Indeed modulation of motor output would have led to potentiation (or suppression) of both types of “go”-responses. Here we build on this observation and extend it to the domain of emotional biasing. In contrast with classic upper-extremity approach-avoidance tasks where forward-backward movement direction is associated with approach-avoidance actions (Rotteveel and Phaf, 2004; Markman and Brendl, 2005), we employed an emotional decision making task, in which, as in Huys et al. (2011), the motor responses are matched for instrumental approach and avoidance. Furthermore, the direction of the emotional response axis (lean forward/freeze/lean backward in response to the emotional faces) and the direction of the instrumental approach/avoidance axis (step left-rightwards) were orthogonalized. Specifically, the instrumental task required participants to learn to step leftwards or rightwards on a balance board, in a manner that corresponded, or was opposite to the location of an instrumental target on the screen. The operationalization of approach-avoidance actions in terms of actual whole-body movements towards/away from a target incidentally also minimized the ambiguity of the experimental definition of approach-avoidance actions, as is the case in classic upper-extremity approach-avoidance tasks (Rotteveel and Phaf, 2004; Markman and Brendl, 2005). In sum, the main purpose of the present study was to test the hypothesis that emotional biasing of action selection reflects effects of a system that controls innately specified responses. Moreover, this system was anticipated to interact with the instrumental action selection system at the level of motivational valence rather than motor output. Thus, we expected the emotional biases to be action-specific, so that angry faces would potentiate instrumental avoidance responses (left- or rightwards steps to the location opposite to that of the instrumental stimulus), and inhibit instrumental approach responses (left- or rightwards steps to the locations corresponding to that of the instrumental stimulus), while the opposite would hold for happy faces. Critically, the strength of this action-specific effect of emotional faces was expected to be related to the strength of the bodily freezing responses.

## Methods

### Participants

Given the gender differences in the processing of angry faces (Rotter and Rotter, 1988), and to reduce between-subject variability, this study was restricted to women. Forty-five female students, 18–28 of age (*M* = 21.8, *SD* = 2.2), from the Radboud University Nijmegen participated in this study after giving written informed consent. They received payment or course credits as a reimbursement for participation. All participants were healthy with normal or corrected-to-normal visual acuity. Exclusion criteria were regular use of medication (except for contraceptives) or use of psychotropic drugs. The study was performed in accordance with the Declaration of Helsinki and approved by the local ethical committee. Data from two participants were excluded from data analyses. One participant failed to complete the experiment due to nausea. The other participant represented an outlier (>3 standard deviations from the mean) on one of our primary measures: the postural sway difference between angry and happy faces. Accordingly, data are reported from 43 participants.

### Experimental setup

**Figure 1:**
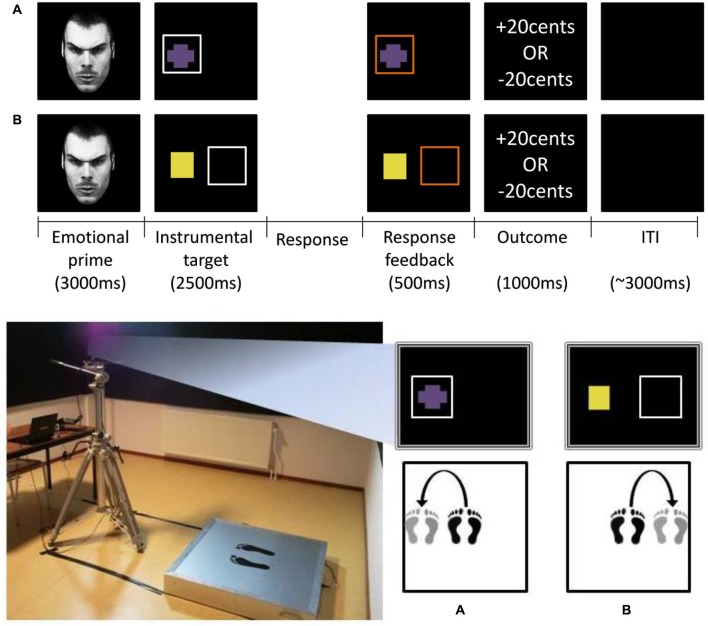
**Top panel: emotional decision making task**. Trial events from the **(A)** approach block and **(B)** avoidance block. After face-prime offset (3000 ms), the instrumental target appeared, to which subjects were required to make a go- or nogo-response within 2500 ms. Response feedback (500 ms) was provided before the monetary outcome (1000 ms). In these examples a go-response had been recorded as indicated by the orange-colored squares during the response feedback phase. The duration of the intertrial interval was 3000 ms on average. **Bottom panel**: Balance board apparatus (left) and examples of a go-response (right) in **(A)** the approach block and **(B)** the avoidance block. Approach-go: sideway step on the balance board towards the side of the instrumental target **(A)**. Avoidance-go: sideway step on the balance board away from the side of the instrumental target **(B)**. Approach-/avoidance- nogo-responses involved remaining stationary at the center of the balance board.

Figure 1 (bottom panel, left) illustrates the experimental set-up. Participants performed the task on a custom-made strain gauge force plate (dimensions: 1 × 1 m; sampling frequency: 100 Hz; 1 mm accuracy), which consisted of four sensors measuring forces in the (vertical) *z*-direction. The signals were used to derive time series of the center of pressure (COP), for the anterior-posterior (AP) and the medio-lateral (ML) direction. Approximately 1 m in front of the participant, visual stimuli were presented at eye height on a 22-inch height-adjustable screen.

### Emotional decision making task

The goal of the paradigm was to assess whether emotional biasing of instrumental action selection would be predicted by innately specified responses, elicited by negative relative to positive emotional stimuli. To this end we employed a probabilistic learning paradigm combined with a balance board apparatus (Figure 1, bottom panel [left]). In this paradigm participants had to learn whole body go- and nogo-responses by trial and error on the basis of monetary outcome (wins/losses of €0.20), while being primed by emotional (angry/happy) faces. The rational for using a probabilistic learning paradigm was to promote recruitment of a model-free habit system, which is thought to be more vulnerable to influences of emotion than is a model- (rule-)based system (Dickinson et al., 1995; Holland, 2004).

The task was framed in terms of a gems collecting and sorting task and consisted of two blocks with different action-contexts (approach/avoidance). These action-contexts determined whether a go-response upon an instrumental target (colored shape, representing a gem) was an active approach- or an active avoidance-action. Thus, there were four types of instrumental responses in total: approach-go, approach-nogo, avoidance-go, avoidance-nogo. We rewarded go- and nogo-responses equally by designating the go-response as the optimal response to half of the instrumental targets. In the approach block, good gems (targets 1–3) were to be approached, whereas bad gems (targets 4–6) were not to be approached. Similarly, bad gems in the avoidance block (targets 7–9) were to be avoided, whereas good gems (targets 10–12) were not to be avoided. As such the value of the four types of instrumental responses was matched. This resulted in a multi-factorial design, in which we manipulated emotional prime (angry/happy), action-context (approach/avoidance), and optimal response (go/nogo) independently (Table 1). Participants were informed that correct choices were reinforced probabilistically. However, they were not informed about the nature of the probabilistic associations.

**Table 1:** **Task-design**.

Action-context	Emotional prime	Optimal response	Probablistic reinforcement: +/- € 0.20
**Approach (120)**			
Instrumental targets:1–3	Angry (30) Happy (30)	Go (60)	*p*(reward|go) = 0.8, *p*(punish|go) = 0.2
Instrumental targets:4–6	Angry (30) Happy (30)	Nogo (60)	*p*(reward|go) = 0.2, *p*(punish|go) = 0.8
**Avoidance (120)**			
Instrumental targets:7–9	Angry (30) Happy (30)	Go (60)	*p*(reward|go) = 0.8, *p*(punish|go) = 0.2
Instrumental targets:10–12	Angry (30) Happy (30)	Nogo (60)	*p*(reward|go) = 0.2, *p*(punish|go) = 0.8

*Emotional prime (angry/happy), action-context (approach/avoidance), and optimal response (go/nogo) were manipulated independently. (*N*)= *N* trials*.

#### Trial events

Figure 1 (top panel) illustrates trial events for (a) the approach block; and (b) the avoidance block respectively. At the beginning of each trial, a task-irrelevant emotional face-prime (angry/happy) was presented centrally on the screen for 3000 ms. Participants were instructed to maintain their view on these stimuli, while remaining stationary. After face-prime offset, the instrumental target appeared either on the left or the right side of the screen, upon which the participant had to make a go- or a nogo-response. If a go-response had not been made within 2500 ms, a nogo-response was recorded. Response feedback in terms of a square (1) turning orange when a go-response was recorded; and (2) remaining white when a nogo-response was recorded, was provided during 500 ms before the monetary outcome (1000 ms). The intertrial interval was jittered (3000 ± 1000 ms). Participants were required to return to their starting position (i.e., the center of the balance board) during the monetary outcome and intertrial interval, if go-response had been made.

Participants were instructed to remain stationary in the center of the balance board for a nogo-response (approach-nogo/avoid-nogo). Importantly, for the go-responses (approach-go/avoidance-go), participants were instructed to make sideway (not forward/backward) steps towards (approach-go) or away from (avoid-go) the side where the gem was presented to approach or avoid the gem respectively (Figure 1, bottom panel [right]). Thus, in contrast with traditional (upper-extremity) approach-avoidance tasks, we ensured that instrumental approach and avoidance required the same motoric “go”-response. Furthermore, the direction of the emotional response axis and the direction of the instrumental approach/avoidance were orthogonalized; that is, the direction of the instrumental response (left-rightward) was orthogonal to the forward-backward movement direction, which has traditionally been associated with approach-avoidance. This more abstract representation of approach-avoidance actions enabled us to address our secondary aim, to disentangle whether Pavlovian-instrumental interaction occurs at the level of motivational valence rather than at the level of motor output: action-specificity (e.g., angry-induced speeding of avoidance, but slowing of approach) would indicate that the interaction occurred at the level of motivational valence rather than motor output. In total, the task consisted of 240 trials. The order of blocks was randomized across participants.

### Visual stimuli

Facial emotional expressions are able to communicate behavioral intentions or action demands to the perceiver (Horstmann, 2003). We have used happy and angry faces, because these emotional faces have been suggested to activate the appetitive (approach) and aversive (avoidance) motivational systems respectively (Roelofs et al., 2009a,b, 2010b; Volman et al., 2011; Von Borries et al., 2012). In contrast with emotional faces such as fear, disgust, sadness, for which the elicited behavioral tendencies may be complex (Marsh et al., 2005; Seidel et al., 2010), happy and angry faces have been shown to induce approach and avoidance behavioral tendencies respectively (Marsh et al., 2005; Roelofs et al., 2009a,b, 2010b; Seidel et al., 2010; Volman et al., 2011; Von Borries et al., 2012).

The face-primes consisted of adult Caucasian faces from 36 models (18 male) from several databases (Ekman and Friesen, 1976; Matsumoto and Ekman, 1988; Lundqvist et al., 1998; Martinez and Benavente, 1998). Model identity was counterbalanced, such that the model occurred equally often for each instrumental target. Each model showed two emotions (angry/happy), matched for brightness and contrast values, displayed against a black background. Faces were trimmed to exclude influence from hair and nonfacial contours (Van Peer et al., 2007). The instrumental targets (gems) consisted of 12 different colored shapes. The emotional primes and the instrumental targets were randomly assigned to each of the two blocks, such that different emotional stimuli and different instrumental stimuli occurred in the two blocks. Stimulus presentation and response acquisition were controlled by a laptop running Presentation software 14.8 12.30.10 (Neurobehavioral Systems, inc.).

### Procedure

Upon arrival, the participants were reminded of the experimental procedure. Subsequently, the participants practiced sideway stepping on the balance board until they felt comfortable with stepping while maintaining their view on the screen. Participants received instructions of the task before each block. To increase ecological validity and participants’ motivation during the task, we told participants that they would receive the total amount of monetary gain as a bonus (on top of the reimbursement).

### Data and statistical analyses

Data-analyses were performed in MATLAB R2009b (The MathWorks, Natick, MA). Statistical analyses were performed using IBM SPSS Statistics 19. We calculated the following parameters: (1) postural mobility (mm/s) for angry vs. happy faces; and (2) reaction time (RT) of instrumental go-responses, and the proportion of instrumental go-responses (P_*go*_) in the emotional decision making task. Repeated measures analysis of variance (ANOVA) were conducted with postural mobility as dependent variable and emotion (angry/happy) as a within-subject variable to assess effects of emotion on bodily freezing. Additionally, two repeated measures ANOVAs with RT and P_go_ as dependent variables were performed with emotion (angry/happy) and action-context (avoidance/approach) as within-subject variables, and postural mobility difference score (D_*postural mobility*_ = postural mobility for angry minus happy) as a covariate of interest to assess whether performance changes on the emotional decision making task were a function of bodily freezing. Significant interaction effects were followed up by simple effects analyses and Pearson correlational analyses. Alpha was set at 0.05.

#### Postural mobility

We operationalized bodily freezing as a reduction in postural mobility for aversive (angry) vs. appetitive (happy) stimuli (D_*postural mobility*_). This operationalization was based on previous studies using posturography to objectively assess human freezing (Carpenter et al., 1999; Azevedo et al., 2005; Stins and Beek, 2007; Roelofs et al., 2010a; Hagenaars et al., 2012, 2014). We quantified postural mobility as the sway path length or the sum of the COP displacements in the AP-ML plane over 1 s in time.

#### Emotional bias in instrumental approach-avoidance

##### Reaction time on go-responses

**Figure 2:**
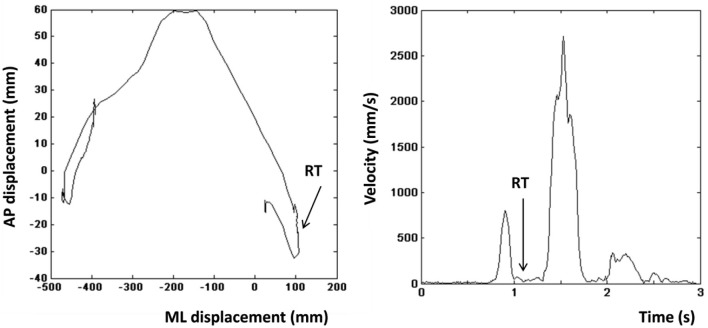
**Left:** Center of pressure (COP) displacement of a representative leftward step. **Right:** Velocity profile of the step shown in the left panel. Arrow indicates the moment that was defined as the reaction time.

Based on previous work (Stins et al., 2011), we calculated the time interval between instrumental stimulus onset and the first overt movement into the intended direction (RT). The left and right panels in Figure 2 show the COP profile of a representative leftward step and the velocity profile of the same step respectively. The step was initiated by lifting the left limb resulting in a brief rightward displacement of the COP (Figure 2, left), which was then followed by a movement of the COP to the left. Directional changes in the COP profile coincide with drops in the velocity profile of the COP (Figure 2, right). The moment, at which the velocity between the first and second peak reaches its minimum, was defined as the RT. Incorrect trials were excluded from RT analyses. Incorrect trials were defined as trials on which participants made a suboptimal or an incorrect instrumental action (approach-go in an avoidance action-context, or avoidance-go in an approach action-context).

##### Proportion go-responses

The proportion of go-responses was calculated by: go/(go+nogo).

## Results

### Postural mobility

ANOVA of the sway path length did not show a main effect of emotion (*F*_(1,42)_ = 0.02, *p* = 0.883, *η*^2^ < 0.001), indicating that on average there was no difference in postural mobility between angry and happy faces.

### Association between bodily freezing and emotional bias in instrumental approach-avoidance

#### Reaction times

**Figure 3:**
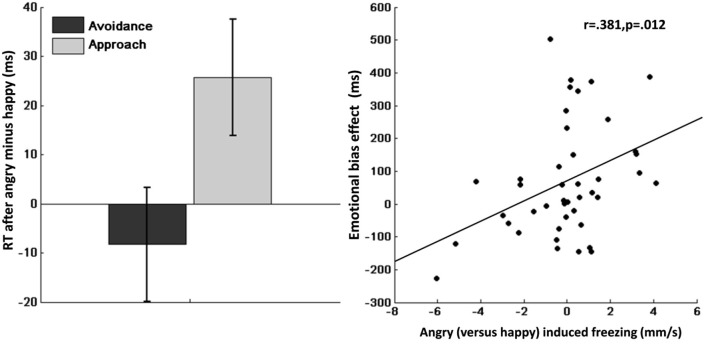
**Left:** Emotional bias effect on instrumental approach-avoidance. Slower approach (vs. avoidance) after angry (vs. happy) faces, showing an action-specific effect of emotional primes. Error bars represent standard error of the mean. **Right:** Correlation between bodily freezing and the emotional bias effect. Angry (vs. happy) induced freezing corresponds to sway (mm/s) during happy minus angry (x-axis). Emotional bias effect corresponds to angry (vs. happy) induced slowing of approach (vs. avoidance) (y-axis).

Mean RTs are shown in Table 2. Repeated measures ANOVA of RTs showed a significant main effect of action (avoidance/approach) indicating faster approach compared with avoidance actions (*F*_(1,41)_ = 19.7, *p* < 0.001, *η*^2^ = 0.32). Moreover, we found a significant emotion (angry/happy) × action-context (avoidance/approach) interaction effect (*F*_(1,41)_ = 8.0, *p* = 0.007, *η*^**2**^ = 0.16), indicating slower approach compared with avoidance after angry vs. happy face-primes (Figure 3, left). Post-hoc analyses suggested that this interaction effect was mainly driven by slower RT after angry vs. happy face-primes in the approach condition (*F*_(1,41)_ = 5.4, *p* = 0.025, *η*^**2**^ = 0.12), but not in the avoidance condition (*F*_(1,41)_ = 0.52, *p* = 0.477, *η*^**2**^ = 0.01).

**Table 2:** **Mean reaction times on the emotional decision making task**.

	Avoidance	Approach
Angry	1215 (24)	1532 (23)
Happy	1223 (24)	1118 (22)

*Reaction time (ms) for instrumental avoidance and approach (SEM) after angry and happy face-primes separately*.

Crucially, we found a significant emotion (angry/happy) × action-context (avoidance/approach) × D_*postural mobility*_ interaction (*F*_(1,41)_ = 7.0, *p* = 0.012, *η*^2^ = 0.15). Correlational analyses indicated that this interaction was explained by an association between reduced postural mobility to angry vs. happy faces and slower RT for approach compared with avoidance after angry vs. happy faces (*r* = 0.381, *p* = 0.012; Figure 3, right). This suggests that individual differences in postural mobility to angry vs. happy faces predict the extent to which angry faces (vs. happy faces) slowed instrumental approach (relative to instrumental avoidance).

Post-hoc analysis suggested that this interaction effect was mainly driven by an association between D_postural mobility_ and the emotional effects on instrumental approach (Emotion × D_postural mobility_; *F*_(1,41)_ = 5.2, *p* = 0.029, *η*^2^ = 0.11). This was reflected by an association between reduced postural mobility to angry vs. happy faces and slower RT for approach after angry vs. happy face-primes (*r* = 0.333, *p* = 0.029), There was no emotion (angry/happy) × D_postural mobility_ interaction in the avoidance action-contect (*F*_(1,41)_ = 0.33, *p* = 0.566, *η*^**2**^ = 0.008).

#### Proportion go-responses

There were no significant main effects and interaction effects for the proportion go-responses (all *F*_(1,42)_ < 2.3, *p* > 0.131, *η*^2^ = 0.05).

## Discussion

In the current study, we combined a novel emotional decision making task with posturography and showed that emotional biasing of action selection reflects effects of a system that controls innately specified responses. We demonstrated that angry vs. happy faces slowed instrumental approach relative to avoidance. Critically, individual differences in this emotional biasing effect were predicted by individual differences in bodily freezing, one of the major innately specified defensive responses to threat (Blanchard et al., 2001). Unlike previous studies on individual differences in motivated behavior, which have often relied on subjective self-report questionnaires (e.g., Carver and White, 1994), we have adopted a mechanistic approach to obtain objective indices of motivated behavior.

The finding that angry vs. happy faces slowed instrumental approach relative to avoidance, demonstrates that the effects of the emotional faces are action-specific. Thus, we extend previous findings by Huys et al. (2011) to the emotional domain by showing that angry vs. happy faces slowed “go”-responses vs. “nogo”-responses related to an approach-action, while having no effect, or, if anything, the opposite effect on an avoidance-action.

A critical finding of the current study is that individual differences in this emotional biasing effect was predicted by individual differences in bodily freezing, one of the major innately specified defensive responses to threat (Blanchard et al., 2001). This finding suggests that emotional biasing of instrumental action involves interaction with a system that controls innately specified responses. This finding is generally in line with contemporary literature stating that behavior depends on multiple systems for decision making (Rangel et al., 2008; Dolan and Dayan, 2013), and more specifically with the idea that emotional anomalies reflect interactions between a Pavlovian and an instrumental control system (Dayan et al., 2006; Seymour and Dolan, 2008). According to this literature, a system that regulates innately specified responses to reward- and punishment-predictive stimuli can bias the instrumental action selection system. Our finding supports this literature, and extends this literature to the emotional domain. Moreover, this finding generally concurs with recent ideas about the survival circuit concept (LeDoux, 2012), which is a concept that integrates ideas about emotion, motivation, reinforcement, and arousal to understand how organisms survive and thrive in daily life. According to this concept, mechanisms that instantiate survival functions, such as a defensive mechanism, are of critical importance in the study of emotional processes.

Research using upper-extremity emotional approach-avoidance tasks has previously shown similar emotion-action interaction effects (also in terms of reaction time; Roelofs et al., 2009a). However, this prior work did not demonstrate that such emotion-action interaction reflects interaction with a system that controls innately specified responses. Moreover, this work did not speak to the degree to which these interaction effects occurred at the level of motivational valence or another level (e.g., motor output). Specifically, in these emotional approach-avoidance tasks, participants are instructed to “approach” and “avoid” visually presented emotional faces by pulling and pushing a joystick. Therefore, the directional axes (forward-backward) of the emotional response and the instructed (instrumental) response overlap. Any modulation of approach/avoidance might thus reflect a competition at the level of motor output rather than a bias at the level of motivational valence. In our current study, the interaction between emotional faces and instrumental action selection was demonstrated when we matched the motor responses for instrumental approach and avoidance, and orthogonalized the directional axes of the emotional approach/avoidance (forward-backward) and instrumental approach/avoidance (left-rightward). Therefore, the present results suggest that the emotional biasing effect occurred at the level of motivational valence rather than at the level of motor output. These findings speak to a traditional debate in the Pavlovian-to-instrumental transfer literature (Lovibond, 1983) about the role of Pavlovian influences, and support the idea that Pavlovian stimuli influence motivational systems that underlie instrumental responding posed by traditional theories on incentive-motivation and two-process theories (Mowrer, 1960; Rescorla and Solomon, 1967; Bindra, 1968; Gray, 1975).

The current study is the first to employ posturography in the investigation of emotional biasing in instrumental approach-avoidance (although see, Gélat et al., 2011; Naugle et al., 2011; Stins et al., 2011, for relevant whole-body movement research on emotional bias in gait initiation). Posturography allowed us to derive more reliable and ecologically valid measures of instrumental approach-avoidance by asking subjects to make actual whole-body movements towards/away from a target incidentally, in contrast with ambiguous experimental categorizations of approach-avoidance in classic upper-extremity approach-avoidance tasks (Rotteveel and Phaf, 2004; Markman and Brendl, 2005). Importantly, this design allowed us to measure bodily freezing (Roelofs et al., 2010a). This implicit and objective measure of an innately specified defensive response to threat allowed us to investigate directly and objectively the degree to which emotional biases on action selection reflect effects of a system that controls innately specified responses, rather than explicit awareness of the experimenters’ demands. Our findings underscore the relevance of integrative emotion, decision making and movement research.

We did not observe a main effect of emotion on postural mobility, i.e., bodily freezing across the whole group. Instead, there was large individual variability, possibly reflecting individual differences in the degree to which angry vs. happy faces elicit defensive behavior; for example in aggressive vs. anxious individuals (Roelofs et al., 2009b, 2010b; Von Borries et al., 2012; see also for a review on the relation between anger and approach motivation, Carver and Harmon-Jones, 2009). Here, we exploit this individual variability in bodily freezing and demonstrate that it predicts the effects of emotional stimuli on instrumental action.

Future studies could benefit from including subjects from different populations, such as patient populations. Our findings may be not only be relevant for understanding the basis of decision making anomalies seen in a wide range of social psychopathology, but also in healthy states, such as fatigue and stress. Particularly, our findings may be interesting in light of recent findings showing that stress may affect systems controlling instrumental action (e.g., Schwabe and Wolf, 2009, 2011). It would be particularly interesting for future studies to assess the interactive effects of stress on the current paradigm. Finally, future studies should test whether these findings can be generalized to men.

In sum, we show that the effects of emotional faces on instrumental responses are action-specific and can occur at the level of motivational valence. Importantly, we found that individual differences in this emotional biasing effect were predicted by individual differences in bodily freezing. This finding suggests that the emotional biasing of action selection by emotional faces reflects effects of a system that controls innately specified responses. Although, this finding is based on correlational analyses, and we have to be careful with causal interpretations, the finding is in support of literature suggesting that behavior depends on multiple decision making systems, an account that may help us understand the mechanisms underlying emotional biasing in decision making. Furthermore, our findings help bridge (animal and human) decision making and emotion research to advance our mechanistic understanding of decision making anomalies in daily encounters as well as in a wide range of psychopathology.

## Authorship

All authors contributed to the study concept and design. Verena Ly was responsible for data-acquisition. Verena Ly analyzed and interpreted the data under the supervision of Karin Roelofs and Roshan Cools, with input by Quentin J. M. Huys and John F. Stins. Verena Ly drafted the manuscript under supervision of Roshan Cools and Karin Roelofs and all others provided critical revisions. All authors approved the final version of the paper prior to submission.

## Funding

This study was supported by the Mosaic grant 017.007.043 from the Netherlands Organization for Scientific Research (NWO) awarded to Verena Ly and VIDI grants from NWO awarded to Karin Roelofs and Roshan Cools, a Human Frontiers Research Grant to Roshan Cools and a James McDonnell Scholar Award to Roshan Cools. Quentin J. M. Huys was supported by the German Research Foundation RA1047/2-1 (Deutsche Forschungsgemeinschaft, DFG).

## Final disclosures

The authors reported no biomedical financial interests or potential conflicts of interest. Roshan Cools is consultant for Abbott laboratories and Pfizer, but not an employee or stock shareholder.

## Conflict of interest statement

The authors declare that the research was conducted in the absence of any commercial or financial relationships that could be construed as a potential conflict of interest.
